# Ophthalmology

**Published:** 1899-06

**Authors:** Alvin A. Hubbell

**Affiliations:** Buffalo, N. Y.


### Progress in Medical Science.

### OPHTHALMOLOGY.

Conducted by ALVIN A. HUBBELL, M. D., Buffalo, N. Y.

### THE SUPERIORITY OF HOLOCAIN OVER COCAINE IN OPHTHALMIC SURGERY.

DR. HASKET DERBY, of Boston, (*Archives of Ophthalmology*, Vol. XVIII., No. 1, 1899,) has used holocain for some time and concludes that in the operation for the extraction of senile cataract it is a most efficient anesthetic. While not superior to cocaine in its superficial effect, it undoubtedly causes a greater degree of insensibility of the iris. Where a simple extraction is not performed and an iridectomy has to be done, we are all familiar with the start the patients may give, as well as the pain they complain of, at this stage of the operation. Under holocain, applied after the corneal cut has been made and the anterior chamber evacuated, it is his experience that the iris very generally allows itself to be seized with the forceps and excised without much if any suffering. This is a very great practical advantage. In connection with the operation of extraction, however, it is but fair to remark on the fact that the holocain does not control hemorrhage as cocaine does, and that where the latter agent is not used we are liable to meet with a troublesome amount of bleeding.

For the removal of a foreign body from the cornea, holocain is decidedly preferable to cocaine, as it neither affects the accommodation nor enlarges the pupil, thus rendering its use possible in the case of people with a tendency to increase of ocular tension. In other operations on the cornea or iris, such as that of Seamisch for ulcus serpens or iridectomy for glaucoma, it is a well known fact that a degree of inflammation that prevents the absorption of cocaine will often yield to holocain, thus rendering the use of ether or chloroform unnecessary. Had cocaine alone been at our command, general anesthesia would have been the only resort.

In the various operations on the muscles of the eye, no local anesthetic has been found to give entire satisfaction. It can only be claimed for holocain in this connection that it is at least as efficient as cocaine, and can be used in cases where distressing constitutional symptoms have been produced by the latter.

In probing the lachrymal passage Dr. Derby still makes a preliminary injection of cocaine, the poisonous effects of holocain, when administered internally, rendering it unsuitable for such a purpose. For the same reason no subcutaneous injection of the drug can be made. But in the numerous cases where he has used it locally and superficially he has never seen the slightest general disturbance.

To sum up, then, the advantages of holocain over cocaine:

1. It does not cause mydriasis, and may therefore be used without danger of bringing about increase of tension.

2. It does not affect the accommodation.

3. It brings about a greater degree of anesthesia of the iris than does cocaine.

4. In cases of severe and painful inflammation which resist cocaine, holocain often proves efficient.

5. Unless swallowed or injected subcutaneously it produces no constitutional effects.

6. It has no effect on the corneal epithelium.

7. It is strongly bactericidal in its action.

Per contra, cocaine distinctly reduces the tendency to hemorrhage and it can be injected into the lachrymal sac, and often subcutaneously, with comparative impunity.

Such being the facts, it would certainly seem that, in the great majority of cases, holocain should supersede cocaine as a local anesthetic in ophthalmic surgery.

---

LOCALISATION VALUE OF OPTIC NEURITIS IN INTRACRANIAL TUMORS.

In a valuable discussion on localisation of the intraocular tumors, recently published (autumn number of *Brain*, 1898,) Marcus Gunn, of London, a most competent observer, says :

The outstanding facts of these investigations and of all clinical experience are, first, the great frequency of optic neuritis as a symptom in connection with tumor; second, the particular proneness of the optic nerve to be affected in tumor of the cerebellum, and after it, in tumor of the basal ganglia, and of the occipital, frontal and tempero-sphenoidal lobes of the cerebrum, these being mentioned here in the order of greater frequency.

Such facts are, however, evidently of practically no localising importance in any particular case. Indeed, common as optic neuritis is in intracranial tumor, we know that a large new growth may exist and be found after death, without any optic neuritis having been present; while, on the other hand, optic neuritis has been diagnosticated during life, without any cause for its occurrences being recognised by highly competent observers at the autopsy.

It is not so much the locality of an intracranial tumor, as its behavior, that influences such symptoms as headache and optic neuritis. Rapid growth or liability to sudden congestion; association with an increase of cerebro-spinal fluid, or a complication with basal meningitis, are the facts that seem to particularly predispose to the occurrence, and afterward to the severity of inflammation of the optic nerve. It is doubtless in consequence of this relation between behavior and intensity, and not from anything particular in the mere position of the new growth, that tumors affecting the cerebellum peculiarly predispose to involve the optic nerve.

Such being the case, is there anything in the character and behavior of the optic neuritis itself—apart from its mere occurrence

—that is of any localising value? I know that I have the support of some of my medical colleagues in believing that cerebellar tumor very often excites a peculiarly intense optic neuritis, with great enlargement of the papillæ, and marked edema of the surrounding retina, with, not uncommonly, a muscular stellate figure, similar to what we get in so-called albuminuric retinitis. This retinal appearance with intense papillitis is in my experience most apt to occur in cerebellar tumor and in basal meningitis—perhaps in cerebellar tumor, because it may so readily cause a basal meningitis. I have met with this form, however, in at least two cases of frontal tumor, in neither of which, I believe, was there any evidence of the basal meninges being involved.

We know that in some cases of optic neuritis, there is a markedly rapid increase in the amount of swelling of the papillæ, presumably due to the concurrent increase of intracranial pressure, for the converse at least is true, that a surgical relief of intracranial pressure is followed by, and doubtless causes, a similarly rapid diminution of disc-prominence. If it can be shown that tumors in certain localities are particularly liable to raise intracranial tension, then this ophthalmoscopic sign would have some localising value, but not otherwise.

And now a few words as to a possible relationship between uniocular optic neuritis and one-sided brain tumor. The explanation of optic neuritis accompanying intracranial tumor is confessedly still obscure, and it is difficult to realise why, on any of the theories advanced, one optic nerve should be attacked and the other long remain normal. But unilateral excess of neuritis may admit of explanation.

1. If optic neuritis be of the nature of a gradually extending inflammation, communicating along the pial sheath of the nerve from the basal meninges, a greater proximity of the exciting tumor to one optic foramen would cause the optic nerve passing through it to be first involved. If this reasoning be correct, in the case of one-sided growth affecting the base far toward the frontal and anterior part of the tempero-sphenoidal lobes, the optic neuritis would be first recognised, and would for some time be in excess, on the same side as the tumor. In the case of a tumor located farther back, the affection of the pia mater would presumably extend forward equally along both nerves after it had reached the chiasma; and in such a case, accordingly, the optic neuritis might be expected to appear practically simultaneously, and be equal in intensity, on the two sides, and that, in any case, it would have no lateral localising significance. A one-sided difference of neuritis proves to be much more common in tumors of the cerebral lobes than in tumors of the cerebellum.

2. On the theory of a *descending cerebritis*, it is impossible to understand how the inflammation can fail to be transmitted along both optic nerves on passing the chiasma, even though it had previously been confined to one optic tract. The same remark applies to the supposed influence of the external geniculate body.

3. If increased intracranial pressure be taken as the determining cause, it is manifest that the actual tension must be the same in all parts of the cranial cavity, and that if the two optic nerves are systematically situated they must be equally exposed to the effects of the pressure. But it is possible that anatomical differences, developmental or pathological, may be responsible under pressure-causation for some of the very rare cases where one optic nerve only is inflamed, and of much more common instances of one nerve being involved in advance or in excess of the other. Minor degrees of want of symmetry of the cranial bones on the two sides are not uncommon. And should the disposition and size of the optic canals be in this way different, it is conceivable that this may determine the escape of one nerve, or a modification of its proclivity to the effects of pressure. But of this form of asymmetry we know nothing exact.

Of want of symmetry farther forward, however, we know much more. It is, I think, a fairly general belief, as was pointed out by Gowers, that optic neuritis is, on the whole, more common in hypermetropic eyes. If, with due regard to the frequency of hypermetropia, this is really true, as it seems to be, then it would be interesting to find how far this may be responsible for unequal optic neuritis. In measuring the swelling of the papilla we always require to estimate the refraction of the eye away from the optic disc, at or near the macula, by preference, and it is customary from these data to compare the height of the disc above the general fundus-level in the two eyes. But it would be important also to compare the refraction of the two eyes. How far such a factor is influential, I am not able at present to say; it is unlikely that it should be important except where the difference in refraction is very decided. Of course, its influence, if actual, would minimise the localising value of unsymmetrical optic neuritis, restricting its applicability to cases where the refraction of the two eyes is the same or nearly so.

On the whole, then, while we all fully recognise the value of the ophthalmoscope in the diagnosis of intracranial tumor, it is necessary to be on our guard not to expect too much from such information, either as to the size of the tumor or its localisation, or disappointment will be the result. Carefully recorded observations, however, may yet teach us much that we wish to know. At present I do not think that we are justified, in respect to this method of localising intracranial growths of saying more than

1. That intense double optic neuritis, with much swelling and surrounding retinal change, coming on quickly, suggest the *cerebellum*.

2. That one-sided optic neuritis, or marked difference, suggests the *cerebrum*, and is, on the whole, in favor of the tumor being on the same side as the excess of neuritis, where there are other reasons for localising one in the front of the cerebrum.

